# Serum Amyloid P Component Ameliorates Neurological Damage Caused by Expressing a Lysozyme Variant in the Central Nervous System of *Drosophila melanogaster*

**DOI:** 10.1371/journal.pone.0159294

**Published:** 2016-07-18

**Authors:** Linda Helmfors, Liza Bergkvist, Ann-Christin Brorsson

**Affiliations:** Division of Molecular Biotechnology, Department of Physics, Chemistry and Biology, Linköping University, Linköping, Sweden; Alexander Fleming Biomedical Sciences Research Center, GREECE

## Abstract

Lysozyme amyloidosis is a hereditary disease in which mutations in the gene coding for lysozyme leads to misfolding and consequently accumulation of amyloid material. To improve understanding of the processes involved we expressed human wild type (WT) lysozyme and the disease-associated variant F57I in the central nervous system (CNS) of a *Drosophila melanogaster* model of lysozyme amyloidosis, with and without co-expression of serum amyloid p component (SAP). SAP is known to be a universal constituent of amyloid deposits and to associate with lysozyme fibrils. There are clear indications that SAP may play an important role in lysozyme amyloidosis, which requires further elucidation. We found that flies expressing the amyloidogenic variant F57I in the CNS have a shorter lifespan than flies expressing WT lysozyme. We also identified apoptotic cells in the brains of F57I flies demonstrating that the flies’ neurological functions are impaired when F57I is expressed in the nerve cells. However, co-expression of SAP in the CNS prevented cell death and restored the F57I flies’ lifespan. Thus, SAP has the apparent ability to protect nerve cells from damage caused by F57I. Furthermore, it was found that co-expression of SAP prevented accumulation of insoluble forms of lysozyme in both WT- and F57I-expressing flies. Our findings suggest that the F57I mutation affects the aggregation process of lysozyme resulting in the formation of cytotoxic species and that SAP is able to prevent cell death in the F57I flies by preventing accumulation of toxic F57I structures.

## Introduction

Lysozyme is a glycosidase that is expressed in granulocytes, monocytes and macrophages. It is abundant in diverse bodily fluids such as tears, breast milk and saliva, where it acts as a first line of defence against bacteria. However, several mutations in the gene encoding the protein, including Y54N, I56T, F57I, W64R, D67H and T70N/D112H (in humans) give rise to lysozyme amyloidosis [1]. *In vitro* studies of amyloidal lysozyme variants suggest that reduction in the native state stability leads to formation of transient, partially unfolded species that can aggregate and form amyloid fibrils [2]. Large amounts of amyloid deposits may occur in vital organs, such as the upper gastrointestinal tract, colon and kidneys [3]. Progression of the disease causes organ failure and ultimately death.

Serum amyloid p component (SAP) is a protein that is produced in liver hepatocytes and circulates in human blood [4]. SAP is a member of the pentraxins, which are characterised by a cyclic pentameric structure and calcium-dependent ligand binding. The major function of pentraxins is to bind microbial pathogens or cellular debris during infection or inflammation and contribute to the clearance of pathogens through the innate immune system via complement activation [5]. More specifically, SAP binds to and stabilises DNA in chromatin that has migrated to the extracellular space due to apoptosis or necrosis, thereby protecting it from degradation [6]. In the absence of SAP, aggressive degradation of exposed chromatin may enhance its immunogenicity [7].

SAP can bind to structures shared by all types of amyloid fibrils [8] and is known to associate with lysozyme fibrils. ^123^I-labelled SAP has even been used for scintigraphic detection of lysozyme deposits in vivo [9]. It has been proposed that SAP binds to all forms of amyloid fibrils, is ubiquitously present in amyloid deposits [10] and prevents proteolytic cleavage by decorating and stabilising the aggregates [11]. It has been subsequently suggested that SAP plays a surveillance role *in vivo*, binding to misfolded species and preventing seeding of larger aggregates [12], and/or that decoration of amyloid fibrils and their pre-aggregated precursors could provide a complementary defence mechanism against the formation of toxic aggregates [13]. Thus, there are clear indications that SAP may play an important role in lysozyme amyloidosis that requires further elucidation.

We have previously established a *Drosophila melanogaster* model for lysozyme amyloidosis to investigate *in vivo* behaviour of disease-associate lysozyme variants using the ubiquitous and retinal drivers Act5C-Gal4 and gmr-Gal4 [14]. In the cited study we showed that expression of the amyloidogenic isoforms results in degradation of the variants and up-regulation of the unfolded protein response (UPR). The UPR is a response to endoplasmic reticulum (ER) stress caused by accumulation of unfolded or misfolded proteins in the ER, mediated through one or more of three main pathways, designated IRE-1, PERK and ATF-6.

The UPR is a key mechanism for restoring ER homeostasis by transcriptionally up-regulating ER chaperones to assist protein folding, attenuating the overall translation rate and increasing the degradation of misfolded proteins in the ER [15,16]. However, whereas transient ER stress can be alleviated by the UPR, severe or prolonged ER stress and up-regulation of UPR can trigger apoptosis [17].

To further elucidate progression of lysozyme amyloidosis, in this study, human WT lysozyme and the disease-associated variant F57I were expressed in the central nervous system (CNS) of *Drosophila melanogaster* with and without co-expression of SAP. The reason for directing the protein expression to the CNS was the possibility to study cytotoxicity using a longevity assay in parallel with accumulation of lysozyme species. This cannot be achieved using the Act5C-Gal4 driver or the gmr-Gal4 driver since high levels of WT lysozyme are lethal during metamorphosis when ubiquitously expressed [14] and the retinal driver is known to be active only until 2 weeks post-eclosion [18]. We examined the longevity, the presence of apoptotic cells, the levels of soluble and insoluble lysozyme and the up-regulation of UPR in the flies. Our findings show that SAP can inhibit apoptosis and restore neurological deficiencies in flies expressing the amyloidogenic variant F57I in the CNS.

## Results

### Co-Expression of SAP Extends the Median Lifespan of F57I-Expressing Flies by 13 Days

In this study we have explored the effects of expressing human wild type (WT) lysozyme and the amyloidogenic variant F57I in the CNS of *Drosophila* with and without co-expression of SAP. Protein expression was driven by a novel nsyb-Gal4 construct, which drives at considerably higher levels than the frequently used elav-Gal4 line, and is restricted to postmitotic neurons [19]. Initially, we examined the lifespan of the flies, which provides a quantitative measure of the toxic effects of expressing amyloidogenic proteins in *Drosophila* CNS [20,21].

**Fig 1:**
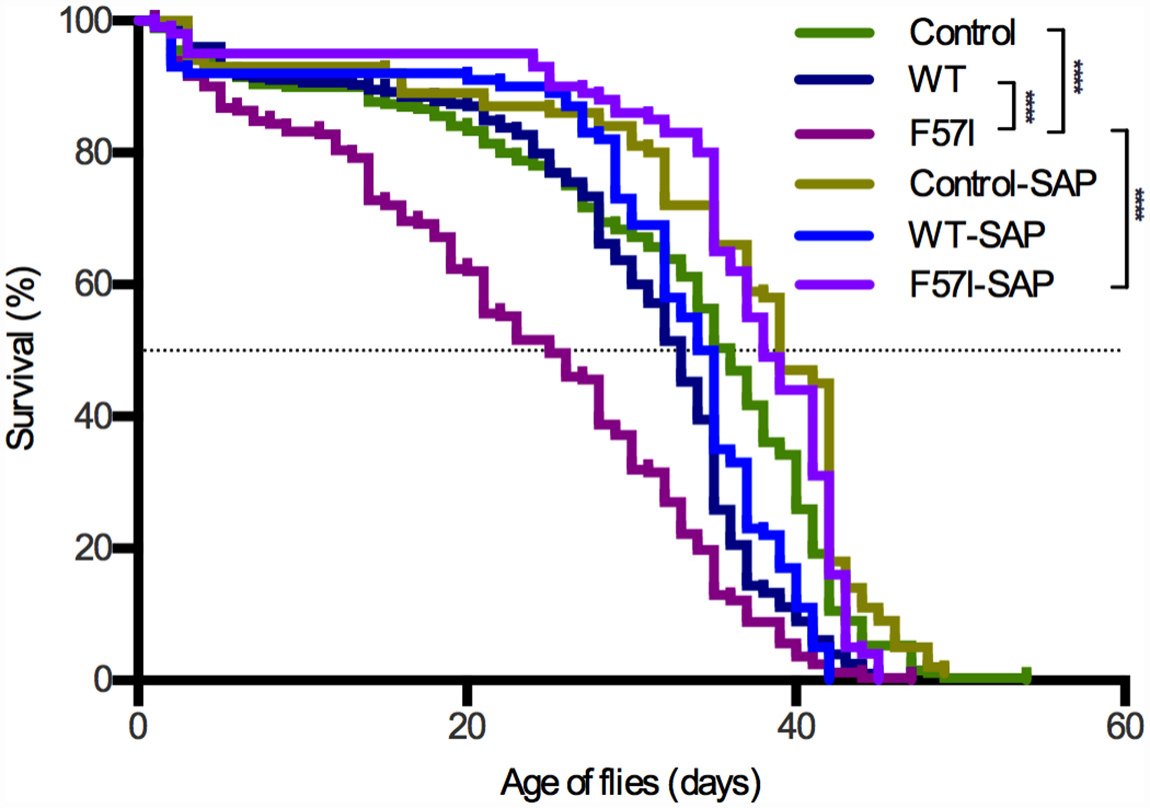
The median lifespan for F57I-expressing flies is extended by 13 days upon co-expression with SAP. Survival trajectories for flies expressing WT lysozyme or the F57I variant in the CNS and for nsyb-Gal4 control flies, with and without co-expression of SAP. Median survival times of control, WT and F57I flies were 36, 33 and 25 days, respectively in the absence of SAP and 39, 35 and 38 days, respectively in the presence of SAP. Kaplan-Meier graph showing per cent survival vs age of flies in days (****p<0.0001).

Median survival times of nsyb-Gal4 background *w*^*1118*^ control flies (which do not express human lysozyme), WT- and F57I-expressing flies were 36, 33 and 25 days, respectively. Thus, flies expressing the amyloidogenic variant F57I had a significantly (p<0.0001) shorter lifespan than those expressing WT lysozyme and control flies (Fig 1). The corresponding survival times with co-expression of SAP were 39, 35 and 38 days, respectively. Thus, SAP significantly (p<0.0001) extended the median lifespan of the F57I flies by 13 days (Fig 1). The result from the longevity assay demonstrates that expression of the F57I variant in the CNS of *Drosophila* has a toxic effect that can be countered by co-expression of SAP.

### Co-Expression of SAP Prevents Neuronal Death in F57I-Expressing Flies

**Fig 2:**
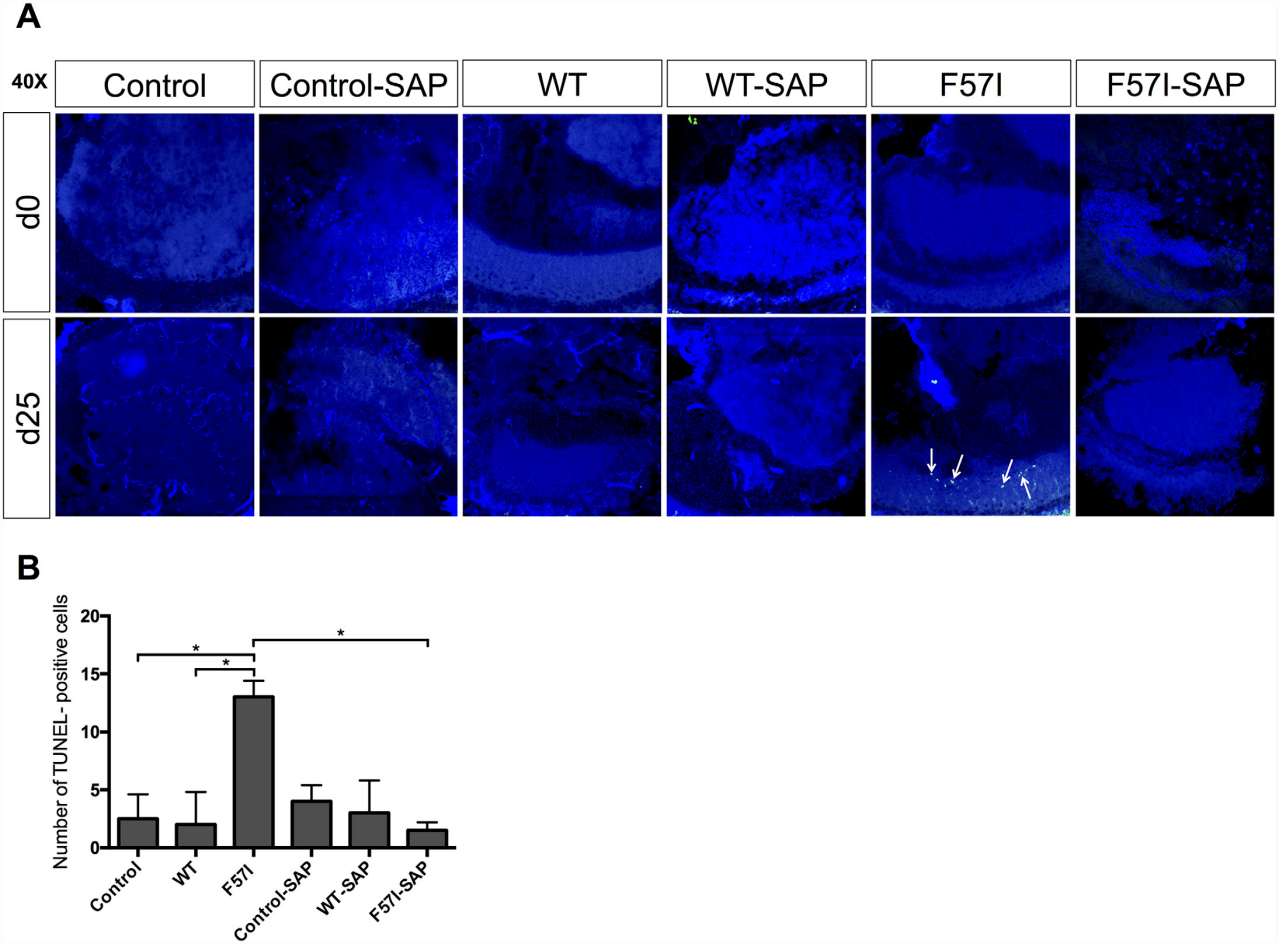
The presence of SAP prevents neuronal cell death in F57I-expressing flies. (A) Apoptotic cells in control, WT and F57I, with and without co-expressing SAP, identified by TUNEL staining at day 0 and day 25. Micrographs were taken at 40x magnification. (B) The number of TUNEL-positive cells in the brains of F57I-expressing flies, collected at day 25, compared with the number of TUNEL-positive cells in age-matched control, WT, control-SAP, WT-SAP and F57I-SAP flies. Bars represent means ± s.e.m, n = 3 brain sections (*p<0.05).

By using the terminal deoxynucleotidyl transferase dUTP nick end labeling (TUNEL) assay, the presence of apoptotic cells in *Drosophila* brains, collected at day 0 and day 25, was investigated for control, WT- and F57I-expressing flies with and without co-expression of SAP. In the absence of SAP, an increase in TUNEL-positive cells was identified in the F57I-expressing flies at day 25 compared to day 0 (Fig 2A). The increase in TUNEL-positive cells reveals the presence of apoptotic cells in aged F57I flies, which occurred at significantly higher numbers compared to WT and control flies at day 25 (p<0.05; Fig 2B). Impressively, the number of TUNEL-positive cells at day 25 in the F57I-expressing flies was significantly reduced in the presence of SAP (p<0.05), demonstrating that SAP is able to inhibit cell death in these flies (Fig 2A and 2B).

### SAP Prevents Accumulation of Insoluble Forms of WT and F57I Lysozymes

To further explore effects of expressing WT and F57I, with and without SAP co-expression in *Drosophila* CNS, we measured lysozyme levels in fly heads collected at day of eclosion (day 0), day 25 and day 35 using an electrochemiluminescent antibody-based protein assay (Meso Scale Discovery (MSD) protein assay). The protein samples were divided into soluble and insoluble fractions to examine the possible presence of insoluble lysozyme aggregates in the flies.

**Fig 3:**
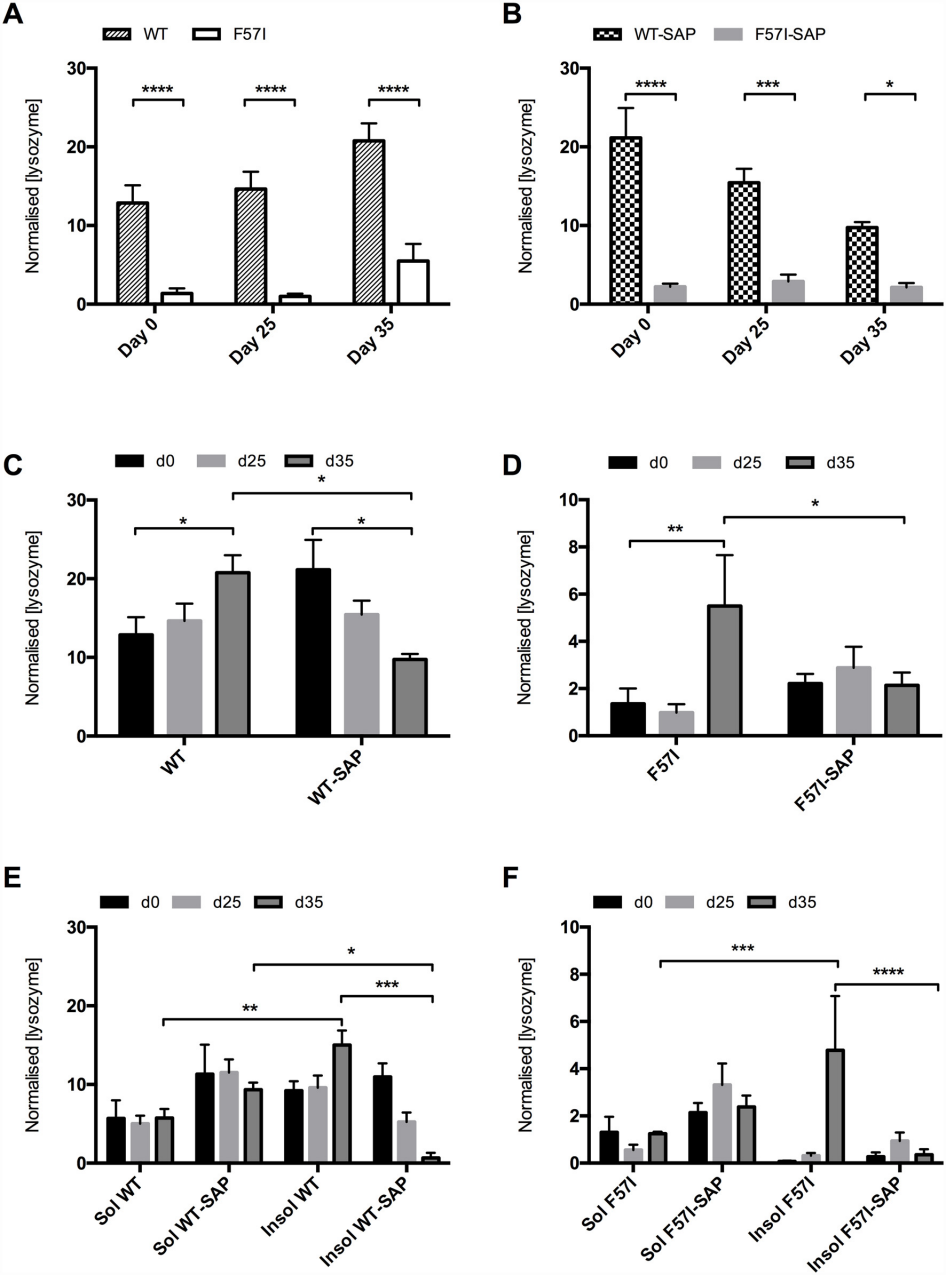
Co-expression with SAP maintains both WT and F57I lysozymes in the soluble fraction. (A) Total amounts of lysozyme in WT and F57I fly samples without SAP co-expression. (B) Total amounts lysozyme in WT and F57I fly samples in the presence of SAP co-expression. (C) Total amount of lysozyme in WT flies both in the absence and presence of SAP. (D) Total amount of lysozyme in F57I flies both in the absence and presence of SAP. (E) Amounts of soluble and insoluble WT protein and (F) amounts of soluble and insoluble F57I protein both in the absence and presence of SAP. Note the different scale on the y-axis for D and F. Normalised values: nanograms of lysozyme detected per unit total protein content (mg/ml) in the samples after subtracting unspecific signals from control flies. Bars represent means ± s.e.m, n = 3 fly samples each containing ten flies (*p<0.05, **p<0.01, ***p<0.001, ****p<0.0001).

Both in the absence and presence of SAP co-expression, the total lysozyme level (including both soluble and insoluble fractions) was significantly higher in flies expressing WT lysozyme than in F57I-expressing flies at all time-points (days 0, 25 and 35; Fig 3A and 3B). For WT-expressing flies, the total lysozyme level increased significantly from day 0 to day 35 in the absence of SAP whilst a significant decrease in the lysozyme level was detected in the presence of SAP (Fig 3C). The total level was very low in the F57I-expressing flies at days 0 and 25, but increased to a substantial level at day 35 (Fig 3D). When co-expressing SAP and F57I, the total lysozyme level was found to be low at all time-points (Fig 3D). For both WT- and F57I-expressing flies, significantly (p<0.05) lower levels of lysozyme were detected at day 35 in the presence of SAP compared to when the lysozyme variants were individually expressed (Fig 3C and 3D).

Next, the fractions of soluble and insoluble lysozyme were analysed. Both in the absence and presence of SAP, the levels of soluble and insoluble lysozyme detected in the WT flies were not significantly different at day 0 (Fig 3E). In the absence of SAP, the level of the insoluble fraction increased over time resulting in a significantly (p<0.01) higher insoluble:soluble ratio at day 35 (Fig 3E) whilst, in the presence of SAP, the level of the insoluble fraction decreased over time resulting in a significantly (p<0.05) lower insoluble:soluble ratio at day 35 (Fig 3E). In the absence of SAP only soluble lysozyme was detected in the F57I flies at day 0 (Fig 3F), but the insoluble fraction increased over time and at day 35 a significantly (p<0.001) higher portion of insoluble F57I protein was detected compared to the soluble fraction (Fig 3F). In the presence of SAP most of the F57I protein was soluble at all three time-points (Fig 3F) and no increase in the insoluble fraction was detected. Thus, for both the WT and F57I flies, the insoluble level of lysozyme detected at day 35 was significantly lower when co-expressing SAP compared to when the protein variants were individually expressed (p<0.001 and p<0.0001 respectively).

Taken together these data show that in the absence of SAP, both WT lysozyme and the F57I variant accumulated as insoluble species and at day 35 most WT and F57I were insoluble. However, in the presence of SAP no accumulation of insoluble species of either variant was detected. Indeed, at day 35 the insoluble fractions of both the WT protein and the F57I isoform were remarkably low and most of the protein was soluble, clearly demonstrating that SAP prevents accumulation of insoluble species for both WT and F57I.

### SAP Co-Localises with Lysozyme in the CNS

**Fig 4:**
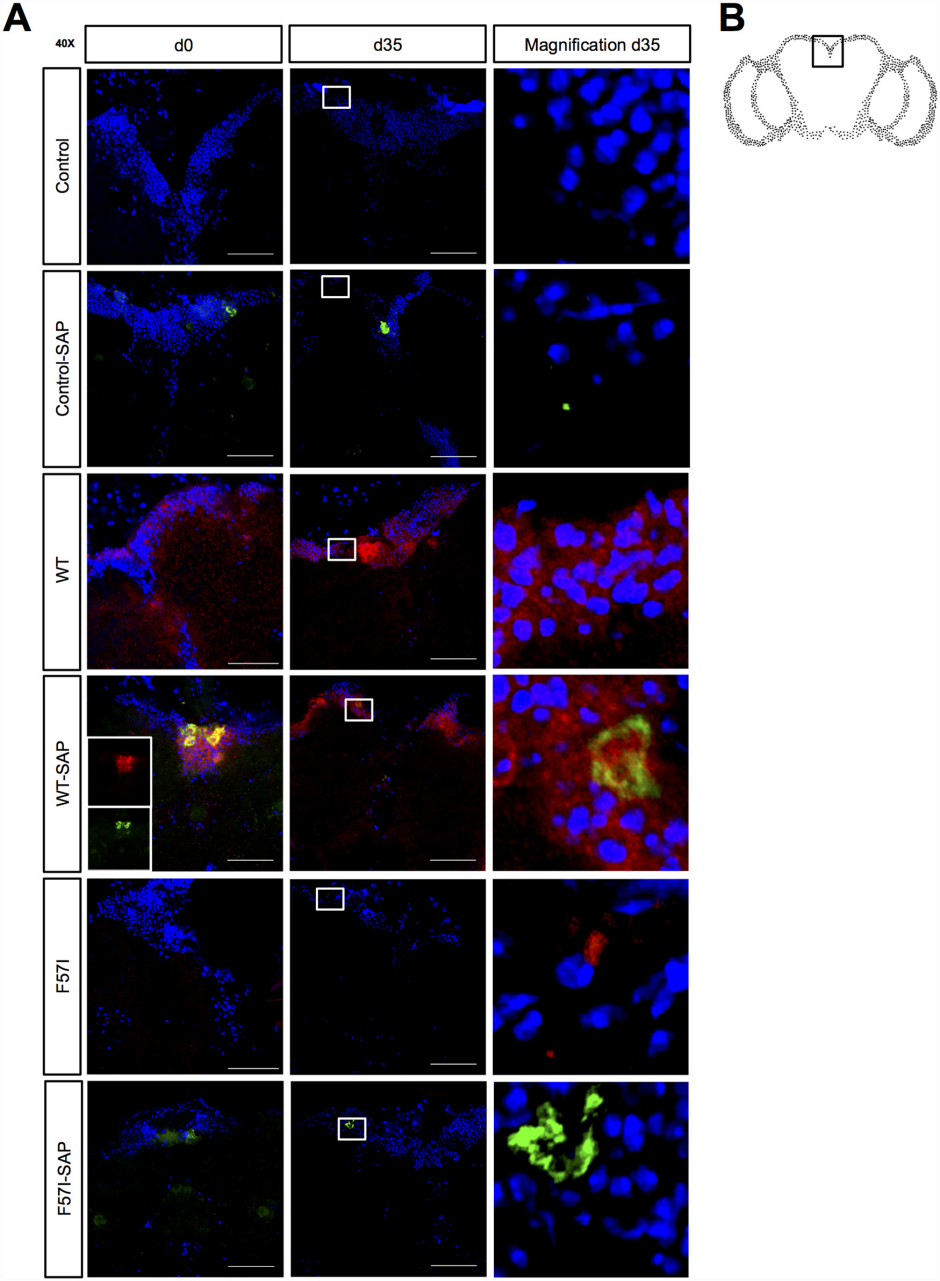
SAP co-localises with WT lysozyme in *Drosophila* CNS. (A) Sections (20 μm) of *Drosophila* brains (day 0 and day 35) stained with an anti-lysozyme antibody or co-stained with both anti-lysozyme and anti-SAP antibodies then counterstained with DAPI. In WT-SAP day 0, inserted images show the SAP (green) and lysozyme (red) channel separately. The white box indicates the area of the brain at day 35 that is shown in the magnification panel. Red and green staining show lysozyme and SAP detected by an anti-lysozyme antibody and an anti-SAP antibody, respectively. Blue shows staining of cell nuclei by DAPI. Co-localisation is shown in yellow. (B) Schematic image of a fly brain indicating which area of the brain that is shown in following micrographs. Micrographs were taken at 40x magnification. Scale bars = 50 μm; n = 7 for all genotypes.

To investigate if lysozyme and SAP co-localise in the CNS, sections of *Drosophila* brains collected at day 0 and day 35 were stained with an anti-lysozyme antibody (WT and F57I flies) or co-stained with anti-lysozyme and anti-SAP antibodies (control, control-SAP, WT-SAP and F57I-SAP flies; Fig 4A) then counterstained with DAPI. The examined area of the fly brain section is specified in the schematic image of a fly brain in Fig 4B. SAP was detected in samples from control-SAP flies, which only express SAP, and in flies co-expressing SAP with WT (WT-SAP) or F57I (F57I-SAP) at both tested days. White box at day 35 (Fig 4A) indicates magnified area of the brain section.

In the WT flies, lysozyme was detected both at day 0 and at day 35 (Fig 4A). The lysozyme signal was increased around the cell bodies and decreased around the neuropils at day 35 compared to day 0 indicating accumulation of WT lysozyme species which goes hand in hand with the result from the protein assay analysis where insoluble species of WT lysozyme were found to accumulate over time (Fig 3E). At day 0, in the WT-SAP samples, almost total co-localisation of SAP with WT was observed (Fig 4A) and structures of approximately 15 μm diameter were detected. These data clearly indicate that SAP interacts with lysozyme in the fly CNS. This co-localization was less pronounced at day 35. At both day 0 and day 35 signals from lysozyme were detected that did not co-localise with SAP.

For flies expressing F57I, no lysozyme was detected in the F57I or F57I-SAP samples at day 0 (Fig 4A), corroborating the findings from the protein assay analysis that these flies had very low levels of lysozyme (Fig 3A and 3B respectively). At day 35, a small lysozyme signal was detected in the F57I samples (Fig 4A) indicating a build-up of F57I species over time in these flies as seen at day 35 in the protein assay analysis (Fig 3D and 3F). In the F57I-SAP samples, no lysozyme was detected at day 35 (Fig 4A) pointing towards the same conclusion as the protein assay analysis, that SAP is able to prevent accumulation of F57I species (Fig 3D and 3F).

### SAP Does Not Affect Up-Regulation of UPR

**Fig 5:**
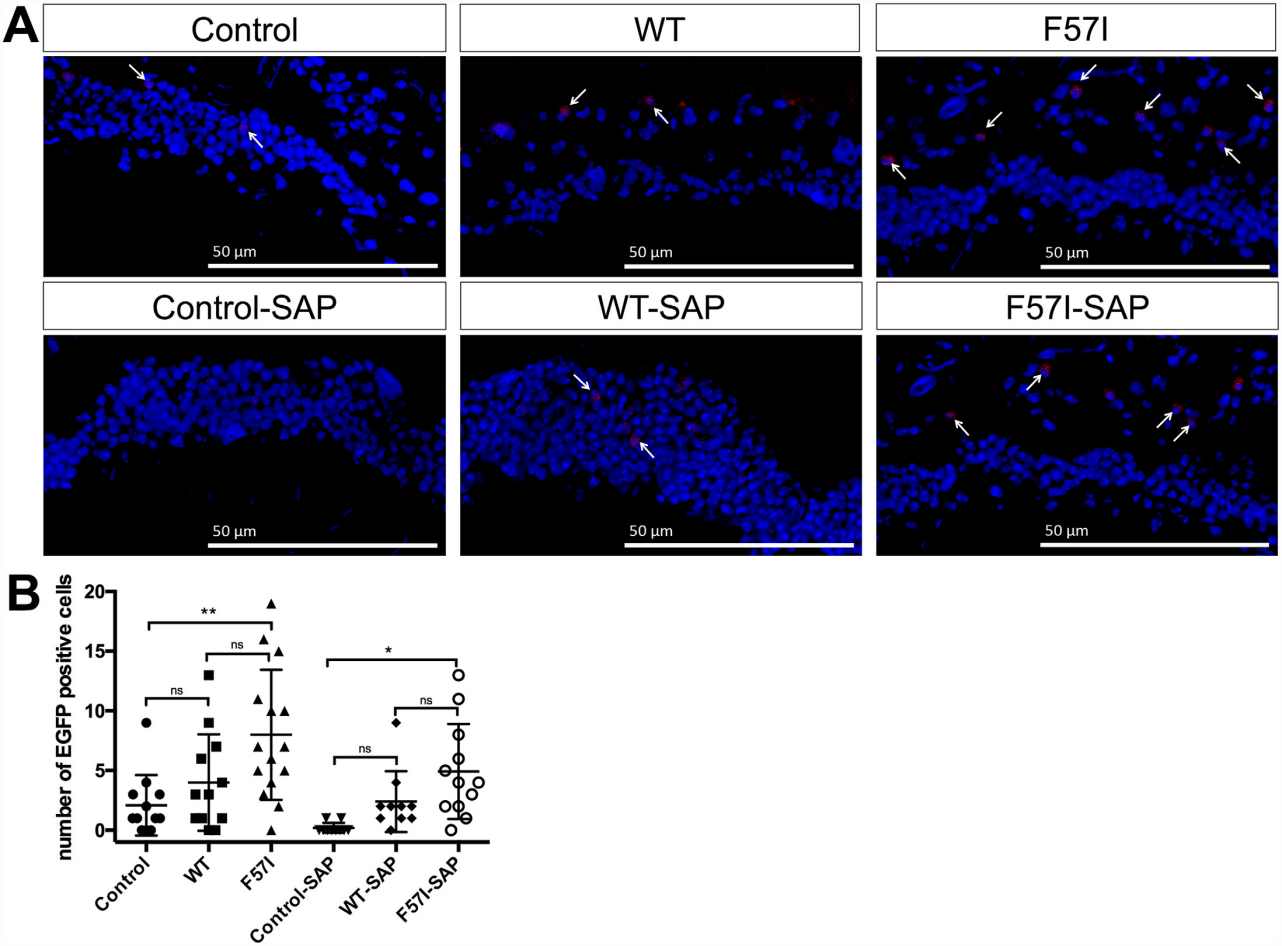
Co-expression of SAP does not prevent UPR activation induced by F57I expression in the fly CNS. (A) Sections (10 μm) of *Drosophila* brains at day 10 stained with anti-EGFP antibody, then counterstained with DAPI. Red stain shows EGFP-positive cells and blue stain corresponds to the cell nuclei. White arrows indicate EGFP-positive cells. (B) Scatter plot shows number of EGFP-positive cells where the micrographs were examined in a blinded experiment, n = 12, 12, 15, 10, 10 and 12 for Control, WT, F57I, Control-SAP, WT-SAP and F57I-SAP, respectively (*p<0.05, **p<0.01). Scale bars = 50 μm.

We have previously demonstrated that expression of the F57I lysozyme variant mediated by the gmr-Gal4 driver triggers UPR up-regulation [14]. To investigate the relationship between UPR up-regulation and expression of lysozyme in *Drosophila* CNS, up-regulation of UPR was quantified in WT- and F57I-expressing flies, with and without SAP co-expression, at day 10 using the xbp1-EGFP reporter, which probes the Ire-1 pathway of UPR up-regulation [15]. When Ire-1 detects ER stress, xbp1 is spliced and EGFP moves into frame resulting in EGFP production. The levels of EGFP in the brain of the flies were determined using immunohistochemistry. Representative micrographs for each genotype can be seen in Fig 5A, white arrows indicate EGFP-positive cells. Both in the absence and presence of SAP, a significant increase in EGFP level was found for the F57I-expressing flies compared to nsyb-GAL4 xbp1-EGFP control flies (p<0.01 and p < 0.05) while no significant difference could be observed between WT-expressing flies and control flies (Fig 5B). The increase in the EGFP level for the F57I and F57I-SAP flies suggests that UPR is up-regulated in these flies. When comparing the EGFP levels between WT- and F57I-expressing flies, with and without SAP, no significant differences were detected. The EGFP levels detected in the WT and F57I flies did not differ in the absence or presence of SAP revealing that SAP does not affect the unfolded protein response in the flies.

## Discussion

We have previously created a *Drosophila* model of lysozyme amyloidosis in which WT lysozyme and amyloidogenic variants were expressed both ubiquitously and solely in the eye [14]. In the cited study we also showed that expression of the amyloidogenic variants resulted in pronounced up-regulation of the UPR and degradation of the proteins. In the study presented here, WT lysozyme and the disease-associated variant F57I were expressed in the *Drosophila* CNS, with and without co-expression of SAP, to refine understanding of the *in vivo* consequences of the amyloidogenic F57I mutation and assess the impact of SAP in our model system of lysozyme amyloidosis.

To determine the overall health of the flies in the absence of SAP we performed a survival assay, which revealed that the F57I-expressing flies had a significantly shorter lifespan than WT and control flies. In Alzheimer’s flies, which express the Aβ peptide in the CNS, reduction in lifespan has been found to be caused by cellular toxicity of the Aβ peptide leading to neuronal cell death [22]. Therefore, our survival data suggested that the neurons of the F57I flies were dying; indeed, by using the TUNEL assay we identified a significantly higher amount of apoptotic cells in the brain of the F57I flies compared the WT and control flies.

Results from the protein level assay show that in the absence of SAP, the lysozyme level was much lower in flies expressing the F57I variant than in WT-expressing flies at days 0, 25 and 35. Data from qRT-PCR assays have shown that transcription levels of the WT and F57I fly lines do not significantly differ [14], thus both fly lines express lysozyme at equal levels. Therefore, the low level of lysozyme found in the F57I flies reveals that a large fraction of the F57I variant is degraded by the quality control system when the protein is expressed in the fly CNS. Our previous data in the above-cited study show that, when expressing the F57I variant in the *Drosophila* eye, the UPR is significantly up-regulated both compared to control flies (only expressing Gal4) and to WT-expressing flies confirming that this amyloidogenic variant is cleared by ER-associated degradation in the *Drosophila* eye. In the present analysis, a significant up-regulation of UPR was detected for the F57I flies compared to control flies suggesting that the low level of lysozyme found in the brain of the F57I flies is due to the protein being degraded by ER-associated degradation. In contrast, no significant up-regulation of UPR was detected for the WT flies compared to control flies suggesting that WT lysozyme is not targeted for degradation by the UPR system resulting in the high levels of lysozyme detected in these flies. These data are in accordance with previous cell based results where only the amyloidogenic lysozyme variants, and not the WT protein, were found to accumulate in the ER resulting in UPR activation [23].

When analysing the levels of soluble and insoluble lysozyme for WT- and F57I-expressing flies it was found that in the absence of SAP, the fraction of insoluble WT species increased over time and insoluble species of the F57I variant also formed over time. These findings were strengthened by the immunohistochemistry results; for WT lysozyme, an immunofluorescence signal was detected both at day 0 and at day 35 where the apparently denser signal around the cell bodies at day 35 suggests accumulation of WT lysozyme species. For the F57I variant, the level of protein was too low to be detected by immunofluorescence at day 0 but at day 35 a clear signal from F57I confirmed accumulation of F57I species over time.

In a recent study [24] the effect on cell viability of different types of WT lysozyme aggregates was examined. The study indicates that depending on the aggregation conditions, aggregation of WT lysozyme can give rise to species with different degrees of cytotoxicity due to intrinsic differences in their physicochemical properties. In our *Drosophila* model neither the insoluble WT species nor intermediate species, formed on the pathway towards insoluble species, are toxic since there was no difference between WT and control flies in longevity and no increase in apoptotic cells was detected. However, the F57I mutation may affect the aggregation process of lysozyme and consequently the physicochemical properties of the formed species in such way that the insoluble F57I species or intermediate species, formed on the pathway towards insoluble species, possess cytotoxic properties and thus contribute to the detected cell death and the reduced lifespan observed for the F57I flies. Indeed, the impact of a single amino acid substitution on the aggregation process has been observed for the Aβ peptide involved in Alzheimer’s disease where the E22G mutation of the Aβ peptide affects the aggregation process resulting in the accumulation of toxic intermediate species responsible for the higher toxic effect exerted by the this Aβ variant compared to the non-mutated Aβ peptide [22,25].

Next, we analysed the effects of co-expressing the lysozyme variants with SAP. A clear rescue effect (p<0.0001) was detected in the longevity assay for the F57I flies where the flies lived 13 days longer when F57I was co-expressed with SAP. In addition, the amount of apoptotic cells detected in the F57I flies by the TUNEL assay was significantly reduced when SAP was co-expressed. These data suggest that SAP can prevent cell toxicity caused by the amyloidogenic variant F57I in the CNS of *Drosophila*.

The protein level assays of flies co-expressing SAP showed that the lysozyme protein level was higher in WT flies than in F57I flies, at all time-points. Thus, a large proportion of the F57I protein expressed is degraded by the quality control system both with and without SAP co-expression. However, in flies co-expressing SAP and lysozyme more WT and F57I remained in the soluble fraction, i.e. the accumulation of insoluble WT and F57I species observed at day 35 in the absence of SAP did not occur in its presence, indicating that SAP can interact with both WT and F57I and prevent accumulation of insoluble species.

To investigate if SAP interacts with lysozyme, both proteins were expressed in the CNS of the flies and probed immunohistochemically. The resulting micrographs at day 0 revealed a high degree of co-localisation between SAP and WT lysozyme, corroborating the hypothesis that SAP interacts with lysozyme *in vivo*. In contrast, only a small fraction of the WT lysozyme co-located with SAP at day 35. Data from the protein level assay revealed that the insoluble fraction of WT lysozyme in the WT-SAP flies was highest at day 0 and substantially reduced at day 35 while the soluble fraction remained constant over time. The presence of SAP clearly hinders accumulation of insoluble lysozyme species. This could be due to SAP recognising insoluble lysozyme species, and promoting degradation of these species and/or that SAP is able to bind to soluble species of lysozyme, promoting their degradation and thereby reducing the pool of lysozyme molecules that can aggregate into insoluble species.

For the F57I-SAP flies no lysozyme was detected at day 0 but more impressively, the lysozyme signal that was found for the F57I flies at day 35 in the absence of SAP cannot be detected at this time when F57I is co-expressed with SAP. This result goes hand in hand with the data from the protein level assay which showed that the accumulated insoluble F57I species detected at day 35 in the absence of SAP does not accumulate in the presence of SAP. Thus, it is likely that SAP is able to recognise insoluble and/or soluble F57I species and promote degradation of these species as suggested for WT lysozyme species. In a recent study it was shown that the extracellular chaperone clusterin is able to interact with oligomeric species of the amyloidogenic lysozyme variant I56T present at low concentration during the lag phase and thereby inhibit its aggregation [26]. SAP has previously been shown to act as a molecular chaperone *in vitro*, enhancing the refolding yield of denatured lactate dehydrogenase and protecting it from inactivation [12]. Thus, it is possible that SAP may act as a molecular chaperone in this *Drosophila* model binding to oligomeric lysozyme species and thereby preventing accumulation of insoluble structures.

As concluded earlier in the discussion, neither the insoluble WT species nor intermediate species formed on the pathway towards insoluble species appear to cause significant neurological damage, since no reductions in the WT flies’ lifespan or increase in neuronal death, relative to those of control flies, were detected. However, if the insoluble F57I species or intermediate species formed on the pathway towards insoluble species are cytotoxic and contribute to the observed cell death and reductions in lifespan of the F57I flies, the ability of SAP to prevent accumulation of these species may contribute to the rescue effects detected in the longevity and TUNEL assays when SAP was co-expressed with F57I.

In other *Drosophila* models, activation of the UPR is commonly protective; for instance its up-regulation protects against neurotoxicity in flies expressing Tau [27] and Aβ amyloid peptides [28]. On the other side, a known effect of UPR activation is up-regulation of apoptosis [29–31] and in our previous study we found up-regulation of UPR in F57I flies and that the eye development was disturbed in these flies [14]. In this study, UPR was found to be equally activated in the F57I and F57I-SAP flies revealing that SAP does not affect the up-regulation of UPR. Thus, it is clear that the decrease in longevity and cell apoptosis observed when the F57I variant is expressed in the fly CNS is not an effect of UPR activation since UPR was up-regulated in the F57I-SAP flies where no toxic effects were detected. This strengthens our hypothesis, that the toxic effects detected in the F57I flies can be caused by formation of cytotoxic F57I species. Taken together, our findings suggest that the F57I mutation affects the aggregation process of lysozyme resulting in the formation of cytotoxic species and that SAP is able to protect nerve cells from damage caused by F57I by preventing accumulation of toxic F57I structures.

We have previously suggested that the onset of lysozyme amyloidosis may be linked to the inability of the UPR to target for degradation the entire population of amyloidogenic variants, allowing a proportion of these species to aggregate and accumulate in the body as amyloid deposits [14]. If we were to speculate regarding the action of SAP in lysozyme amyloidosis, it is possible that SAP is able to bind a portion of the amyloidogenic variants and thereby hinder aggregation and accumulation of these disease-associated lysozymes, possibly by facilitating ER-associated degradation these variants. However, over time a substantial amount of unstable lysozyme variants is able to escape degradation by the UPR pathway allowing them to aggregate and give rise to the disease. Clearly, SAP’s role in amyloid diseases needs to be further investigated.

## Materials and Methods

### *Drosophila* Stocks

The Gal4/UAS system was used for tissue-specific expression of UAS transgenes in *Drosophila melanogaster* [32]. To express the transgenes in the CNS, we used a novel pan neuronal driver (nsyb-Gal4) graciously provided by S. Thor. For xbp1-nsyb-Gal4 flies, xbp1-Gal4, used in previous work [14], was crossed into the nsyb-Gal4 fly line. Transgenic flies containing the genes encoding WT lysozyme and the F57I variant were previously generated [14] and the transgenic SAP flies [13] were kindly proved by E. Lundgren. Strains of nsyb-Gal4 flies carrying UAS containing genes encoding WT, F57I, WT-SAP and F57I-SAP were created. Background nsyb-Gal4 *w*^*1118*^ with and without expression of SAP was used as control flies. Flies were reared at 29°C, 60% RH with 12:12 hour light:dark cycles. Offspring were collected at day of eclosion and transferred either into an Eppendorf tube and snap-frozen for protein assays, or into a 50 ml plastic vial with 6 ml agar food (20 g agar, 20 g sugar, per 1 L water) and yeast paste (dry baker’s yeast mixed with water) for ageing 25 and 35 days. The flies were transferred into a fresh agar tube every 2–3 days.

### Longevity Assay

Sets of 150 flies of each line were divided into plastic vials containing agar food and yeast paste with 10 flies in each vial. Every 2–3 days the flies were transferred to fresh food and the numbers of live flies were counted. The assay was repeated twice and the data were pooled and analysed together. Prism GraphPad software 6 (GraphPad Software, San Diego, CA, USA) was used to generate Kaplan-Meier survival curves [27] and run the log rank statistical analysis.

### TUNEL Assay

*Drosophila* heads were embedded at day 0 and day 25 in Tissue-Tek OCT compound (Histolab, Göteborg, Sweden) using Cryomold-specimen molds and stored at -80°C until use. The OCT blocks were sectioned using a Microm HM 550 Cryostat (Microm International GmbH, Walldorf, Germany) into 10 μm thin sections that were placed on Superfrost Plus slides (Menzel-Gläser, Braunschweig, Germany) and stored at -20°C until use. The TUNEL assay was performed using FragEL^™^ DNA Fragmentation Detection Kit, Fluorescent—TdT Enzyme (QIA39, Millipore). The sections were fixed in 4% (w/v) PFA for 15 min at room temperature (RT), followed by 15 min washing in 1X TBS, RT. The sections were then incubated with 20 μg/ml Proteinase K (JA1477, Millipore) diluted in 10 mM Tris, pH 8 for no longer than 10 min at RT. The slides were then dipped in TBS three times as a washing step. The 5X TdT Equilibration Buffer (JA1748, Millipore) was diluted in dH_2_O and added to the slides for approximately 20 min of incubation, RT. Meanwhile, the TdT Enzyme (A1560; Millipore) was diluted 30 times in Fluorescein-FragEL^™^ Labeling reaction mix (JA1834, Millipore). The TdT Equilibration Buffer was carefully blotted of the slides and the sections were incubated with the diluted TdT Enzyme in 37°C for 75 min in a humid chamber. The slides were then washed three times in TBS, dipped in dH_2_O and left to dry for 1 h at RT. Mounting of the slides was done using Fluorescent Mounting Media (JA1750, Millipore). The slides were then left at 4°C over night, and sealed with nail polish the following day. The slides were analysed using a Zeiss LSM 780 confocal microscope (Zeiss). Micrographs were processed in Adobe Photoshop (Adobe Systems); background levels were reduced and the signal levels were enhanced. All images were treated identically. Quantification of TUNEL-positive cells was performed by visually examining serial frontal sections spanning the entire fly brain. The data were analysed using IBM SPSS Statistics for Macintosh, Version 23.0, using a one-way ANOVA followed by post hoc (Tukey) to identify differences among groups; n = 3 for all genotypes.

### Protein Assay of Lysozyme Levels

The Meso Scale Discovery (MSD) protein assay is an alternative to the traditional ELISA protein assay and uses electrochemiluminescence as the detected signal.

Ten fly heads of each genotype were placed in Eppendorf tubes and snap-frozen. To each tube 150 μl of PBS buffer with Protease Inhibitor (Complete EDTA-free Protease Inhibitor Cocktail Tablets, Roche Diagnostics), PBS-PI, was added and the flies were homogenised using a pestle and vortex. The samples were centrifuged (13000 rpm, 1 min) and the collected supernatant constituted the soluble fraction. An additional 150 μl of PBS-PI was added, the samples were homogenised, centrifuged again and the supernatant was pooled with the soluble protein fraction. The pellet was washed in 200 μl PBS-PI, centrifuged again and the supernatant was discarded. The pellet was re-suspended in 60 μl of urea-extraction buffer (25 mM Tris, 1 mM EDTA, 1% SDS, 8 M urea) and incubated for 5 min, centrifuged (13000 rpm, 2 min) and the supernatant that was collected in new tubes constituted the insoluble protein fraction. A standard binding 96-well multi-array plate (L15XA-6, Meso Scale Discovery, Rockville, MD, USA) was coated with 25 μl of 15 μg/ml cAbHuL6 (the N-terminal domain of a camelid heavy-chain antibody specific for human lysozyme; [28]) and incubated for 1 hour at RT with gentle agitation followed by three washes with 150 μl PBS-T. Blocking solution (25 μl; 1% milk in PBS-T, i.e. PBS with 0.05% v/v Tween-20) was added to the wells and the plate was incubated (1 hour, RT, with gentle agitation). For each sample of fly homogenate, 25 μl was added in triplicate to the wells and incubated (1 hour, RT, with gentle agitation). The plate was washed and 25 μl of rabbit anti-human lysozyme antiserum (diluted 1:1000 in 1% milk in PBS-T) was added to the wells and incubated (1 hour, RT, with gentle agitation). The plate was washed and 25 μl of Sulfo-Tag goat anti-rabbit antibody (R32AB-5, Meso Scale Discovery, Rockville, MD, USA, diluted 1:500 in 1% milk in PBS-T) was added to the wells and the plate was incubated (1 hour, RT, with gentle agitation). The plate was washed a final time and 150 μl of 2X read buffer (R92TC-2, Meso Scale Discovery, Rockville, MD, USA) was added. The plate was analysed using a SECTOR Imager 2400 instrument (Meso Scale Discovery, Rockville, MD, USA). To adjust for variation in the protein extraction step the total amount of protein from each sample of fly homogenate was quantified using a Bio-Rad DC Protein Assay Kit II (500–0112, Bio-Rad, CA, USA). For each genotype, the values obtained from the wells of three plates were entered into GraphPad Prism 6 (GraphPad Software, San Diego, CA, USA), outliers were identified using the built-in function and removed from the data set. The data were then analysed using 2-way ANOVA followed by an uncorrected Fischer LSD test, n = 3, with ten flies in each, for all genotypes.

### Staining for Lysozyme and SAP or EGFP-Positive Cells

*Drosophila* heads were embedded on the day of eclosion or after having been aged for 10 or 35 days in Tissue-Tek OCT compound (Histolab, Göteborg, Sweden) using Cryomold-specimen moulds and stored at -80°C until use. Sections (20 μm for lysozyme and SAP staining, 10 μm for EGFP-positive staining) were taken from the OCT blocks using a Microm HM 550 Cryostat (Microm International GmbH, Walldorf, Germany), placed on Superfrost Plus slides (Menzel-Gläser, Braunschweig, Germany) and stored at -20°C until use. The sections were fixed in 4% w/v PFA for 10 min at RT and the slides were washed in PBS (3 x 5 min) followed by PBS-T (1 x 3 min) before blocking in 10% w/v BSA in PBS-T for 1 hour at RT. Mouse anti-lysozyme antibody (1:100 in 1% w/v BSA in PBS-T; Abcam (ab36362), Cambridge, United Kingdom) and rabbit anti-SAP antibody (1:100 in 1% w/v BSA in PBS-T; Abcam (ab45151), Cambridge, United Kingdom) or, for EGFP-positive staining, mouse anti-GFP antibody (1:1000 in 1% w/v BSA in PBS-T; Abcam (ab1218), Cambridge, United Kingdom) were added to the slides, which were then incubated overnight at 4°C. The slides were subsequently washed with PBS-T (3 x 5 min). Fluorescently-labelled secondary antibodies, goat-anti-mouse and goat-anti-rabbit, (1:600 in 1% w/v BSA in PBS-T; Alexa fluor 488 and Alexa 594; Life Technologies) were applied to the slides, which were then incubated for 1 hour at RT. The slides were washed with PBS-T (3 x 5 min), dipped in dH_2_O and allowed to dry for a couple of minutes in the dark. The slides were mounted using Vectashield with DAPI (Vectro Laboratories, Peterborough, UK), sealed with nail polish and photographed using a LSM 780 confocal microscope (Zeiss). Micrographs were processed in Photoshop (Adobe Systems); background levels were reduced, the signal levels were enhanced and scale bars were added. All images were treated identically. The data were plotted in Graph Pad Prism and analysed using IBM SPSS Statistics for Macintosh, Version 23.0.; n = 7 for all genotypes stained for lysozyme and SAP, n = 12, 12, 15, 10, 10 and 12 for Control, WT, F57I, Control-SAP, WT-SAP and F57I-SAP, respectively, for EGFP-positive staining. The area of the brain sections showing the highest EGFP-signal for all genotypes where chosen for quantification (ganglion opticum medium and internum).

### Statistical Analysis

The data were analysed using IBM SPSS Statistics for Macintosh, Version 23.0 or GraphPad Software. One-way ANOVA followed by a post hoc test (Tukey) was used to identify differences in TUNEL-positive cells. To analyse protein levels, a 2-way ANOVA followed by an uncorrected Fischer LSD test to identify differences among groups was used. For the statistical analyses of EGFP-positive cells, the counting of positive cells was done blinded. The data was then plotted in Graph Pad Prism and analysed in SPSS, using a one-way ANOVA followed by a post hoc test (Tukey) to identify differences among the groups. GraphPad Prism software 6 was used to generate Kaplan-Meier survival curves.
